# Transanal approach for intersphincteric resection of rectal cancer in a patient with a huge prostatic hypertrophy

**DOI:** 10.1007/s13691-016-0272-8

**Published:** 2016-11-24

**Authors:** Yoshiyuki Kiyasu, Kenji Kawada, Kyoichi Hashimoto, Ryo Takahashi, Koya Hida, Yoshiharu Sakai

**Affiliations:** Department of Surgery, Graduate School of Medicine, Kyoto University, 54 Shogoin- Kawara-cho, Sakyo-ku, Kyoto, 606-8507 Japan

**Keywords:** Transanal approach, Total mesorectal excision, Intersphincteric resection, Rectal cancer, Prostatic hypertrophy

## Abstract

The gold standard of surgical technique for rectal cancer is total mesorectal excision (TME). Laparoscopic TME has been proven to provide surgical safety and oncological outcomes equivalent to open TME. However, dissection of the lower rectum has some inherent difficulties related to a narrow pelvic space. The challenge of TME in the lower rectum was confirmed by the Colorectal Cancer Laparoscopic or Open Resection (COLOR) II trial showing a 9% positive circumferential margin (CRM) rate in laparoscopic TME and a 22% positive CRM rate in open TME. Recently, transanal TME has attracted intense attention as a promising alternative to laparoscopic TME. In this video article, we show the performance of a transanal approach for intersphincteric resection (ISR) of rectal cancer in a patient with a huge prostatic hypertrophy.

**Electronic supplementary material:**

The online version of this article (doi:10.1007/s13691-016-0272-8) contains supplementary material, which is available to authorized users.

## Introduction

The gold standard of surgical technique for rectal cancer is total mesorectal excision (TME) [1]. Laparoscopic TME has been proven to provide surgical safety and oncological outcomes equivalent to open TME [2, 3]. However, dissection of the lower rectum has some inherent difficulties related to a narrow pelvic space. The challenge of TME in the lower rectum was confirmed by the Colorectal Cancer Laparoscopic or Open Resection (COLOR) II trial showing a 9% positive circumferential margin (CRM) rate in laparoscopic TME and a 22% positive CRM rate in open TME [2]. Recently, transanal TME has attracted intense attention as a promising alternative to laparoscopic TME [4–8]. In this video article, we show the performance of a transanal approach for intersphincteric resection (ISR) of rectal cancer in a patient with a huge prostatic hypertrophy.

## Case presentation

**Fig. 1:**
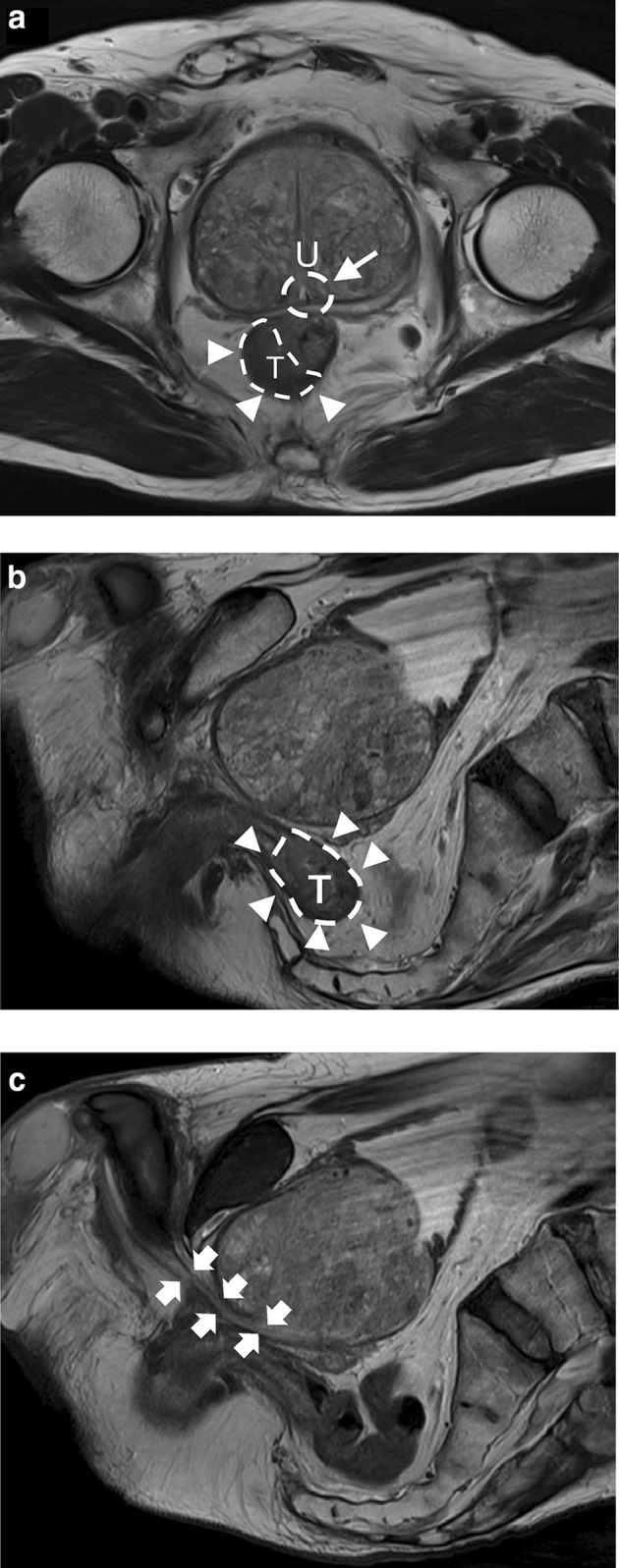
T2-weighted image of MRI. **a** Axial section. The urethra (*white arrow*) was just above the tumor (*white arrowhead*). *T* tumor, *U* urethra. **b** Midline sagittal section. The tumor (*white arrowhead*) was located 3 cm above the anal verge. *T* tumor. **c** Sub midline sagittal section. The urethra (*white arrow*) was deviated by a huge prostate

An 80-year-old man was admitted to our hospital for the treatment of a rectal tumor found incidentally by rectal examination. The tumor, about 3 cm in diameter, was located on the right side of the lower rectum 3 cm above the anal verge. The pathological analysis of the biopsy sample revealed that the tumor was a moderately differentiated adenocarcinoma. Abdominal computed tomography (CT) and magnetic resonance imaging (MRI) indicated that the rectal cancer invaded into the muscularis propria without distant metastases and that lateral pelvic lymph node (LPLN) was not enlarged with a maximum long-axis diameter <3 mm. The most important problem was that the patient had a huge benign prostatic hypertrophy, the size of which was 85 × 80 × 70 mm (Fig. 1a–c). To achieve complete TME with negative CRM, a hybrid transabdominal-transanal approach for ISR was conducted.

### Technique

First, vascular division and mobilization of the left colon were performed laparoscopically. The transabdominal approach was continued until the anterior dissection of the rectum became difficult due to a huge prostatic hypertrophy. Next, the circumferential rectal incision and subsequent intersphincteric dissection were performed under direct vision to enable attachment of a single port device (GelPoint Mini; Applied Medical). After closure of the anal orifice, the GelPoint Mini was placed to start the transanal approach. Posterior side of the rectum was first dissected until the transanal approach was connected to the dissection layer made by the transabdominal approach. The dissection procedure was extended to the lateral side. Bilateral pelvic splanchnic nerves were identified at the 5 and 7 o’clock positions. At the anterior side, the proper dissection layer cannot be easily identified because of the perineal body and the enlarged prostate. Once the dissection plane between the rectum and the prostate could be identified, it was relatively easy to continue along the same plane. The assistance provided by the laparoscopic approach was useful to determine the appropriate dissection line in the transanal approach.

## Result

**Fig. 2:**
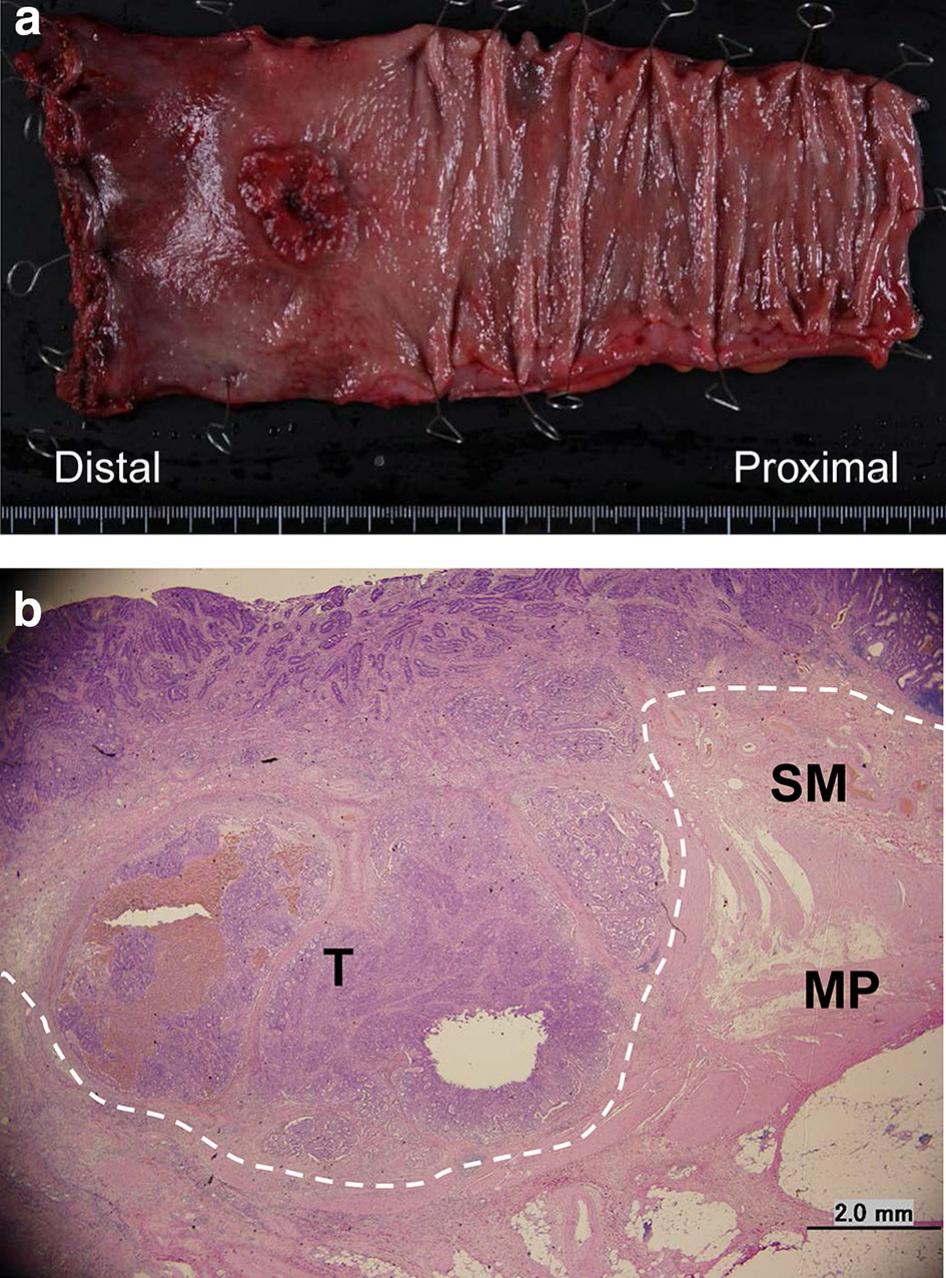
Macroscopic and histolopathological findings of the specimen. **a** Macroscopic finding. The size of the tumor was 25 × 25 mm in size. The distal margin was 40 mm. **b** Histolopathological analysis revealed that the tumor was a moderately differentiated adenocarcinoma (Stage I: T2 (MP), N0 (0/9), and M0 according to the 7th edition UICC). The free distance of CRM was 3000 μm. The *dotted line* the horizontal margin of the tumor, *T* tumor, *SM* submucosa, *MP* muscular propria

The total operative time was 491 min, and the blood loss was 116 ml. Macroscopic findings of the resected specimen showed a solid tumor, 25 × 25 mm in size, and the distal margin was 40 mm (Fig. 2a). On histopathological analysis, the tumor staging was Stage I (pT2N0M0 according to the 7th edition UICC) and the CRM was negative (the free distance of the CRM was 3000 μm) (Fig. 2b). LPLN dissection was not performed, because the indication for LPLN dissection in our institution was defined as LPLN with a short-axis diameter ≥5 mm.

## Discussion

Transanal TME is not well-established technically, but it has potential advantages, including superior visualization, facilitation of TME of the lower rectum, and shorter surgical time and less morbidity [4–8]. The factors that can make transanal TME a preferred approach are (1) male sex, (2) very low location (less than 12 cm from the anal verge), (3) narrow and deep pelvis, (4) visceral obesity (BMI > 30 kg/m^2^), (5) prostatic hypertrophy, (6) large tumor (>4 cm in diameter), (7) distorted tissue planes due to preoperative radiotherapy, and (8) impalpable, low primary tumor [6]. The hybrid approach composed of both transanal and conventional laparoscopic TME can be an especially appropriate choice for difficult cases, such as a huge tumor occupied within a very narrow pelvic space. In this case, extremely huge prostatic hypertrophy made it difficult to determine the appropriate dissection line of TME, especially on the anterior side of the rectum. Transanal TME with the assistance of the conventional laparoscopic approach is very useful to identify the correct TME plane for rectal cancer patients with prostatic hypertrophy.

## Electronic supplementary material

Below is the link to the electronic supplementary material.

Supplementary material 1 (MP4 96006 kb)
